# Renoprotective Effect of Egyptian Cape Gooseberry Fruit (*Physalis peruviana* L.) against Acute Renal Injury in Rats

**DOI:** 10.1155/2014/273870

**Published:** 2014-03-16

**Authors:** Lamiaa Ali Ahmed

**Affiliations:** Nutrition and Food Science Department, Faculty of Home Economics, Helwan University, 65 Elmatbaea El-Ahliaa Street, Boulak Abo Elela, P.O. Box 11611, Cairo, Egypt

## Abstract

This study aimed to evaluate the renoprotective effect of *Physalis peruviana* L. extract (PPE) on acute renal injury in rats. Adult male rats (*n* = 36) were divided into six groups that were fed with basal diet throughout the experiment (33 days). The first group was normal group, the second and the third groups were administered orally with 100 and 150 mg PPE/kg body weight (BW) respectively, the fourth group was injected intraperitoneally with 5 mg/kg BW cisplatin once on the 28th day to induced ARI, and the fifth and sixth groups were treated like the second and the third groups and were injected with cisplatin on the 28th day. Many bioactive compounds were found in PPE. PPE did not cause any changes in the second and third groups compared to normal control group. Administration of PPE prior to cisplatin injection caused significant reduction in relative kidney weight, serum creatinine, urea, blood urea nitrogen, and significant increments in body weight, feed intake, total protein, albumin, and total globulin compared to cisplatin group. Pretreatment with PPE improved kidney histology and diminished the level of thiobarbituric acid reactive substances and enhanced other antioxidant enzymes in kidney homogenate compared to cisplatin group.

## 1. Introduction

Reactive oxygen species (ROS) play a key role in the pathophysiological processes of renal diseases. The cellular damage is mediated by an alteration in the antioxidant status, which increases the concentration of ROS in the stationary state (oxidative stress). Oxidative stress mediates a wide range of renal impairments, from acute renal failure, rhabdomyolysis, obstructive nephropathy, hyperlipidemia, and glomerular damage to chronic renal failure and hemodialysis. Therefore, interventions favoring the scavenging and/or depuration of ROS (dietary and pharmacological antioxidants) should attenuate or prevent the oxidative stress, thereby mitigating against the subsequent renal damage [1].

Cisplatin is a powerful drug for the treatment of many types of cancer [2]. Despite the ability of cisplatin to treat a variety of diseases, its toxicity can result in acute renal failure [3]. Direct tubular toxicity, inflammation, vascular factors, and oxidative stress are the resultant outcome of cisplatin-induced nephrotoxicity [4].

Berries have been shown to provide significant health benefits because of their high antioxidants, vitamins, minerals, and fiber [5]. Cape gooseberry (*Physalis peruviana* L.), known locally in Egypt as harankash and known in English speaking countries as cape gooseberry or goldenberry, hasmany medicinal and edible uses as a promising fruit [6, 7].

The benefits associated with the consumption of* Physalis peruviana* L. (PP) are mainly due to their nutritional composition. It contains biologically active components that provide health benefits and reduce risk for certain diseases. Among its major components are its high amounts of polyunsaturated fatty acids, *β*-carotene, vitamins A, B, and C and phytosterols, and the presence of essential minerals such as iron, vitamins such as E and K1, and withanolides [8, 9].

*Physalis peruviana* L. extracts showed antioxidant activity [10], as well as antihepatotoxic effect [11], antiproliferative effects on hepatoma cells [12], and anti-inflammatory activity [13]. In addition, it has excellent potential as a food-based strategy as antidiabetes and antihypertension solutions [14].

Despite its importance, it remains unknown if PP can possess renoprotective activity against acute renal injury or not. Thus, the purpose of the current study was to investigate whether oral administration of ethanolic extract of* Physalis peruviana* L. fruit has any protective effect against cisplatin induced acute renal injury (ARI) in adult rats.

## 2. Materials and Methods

### 2.1. Preparation of* Physalis peruviana* L. Ethanolic Extract (PPE)

The fruit* Physalis peruviana* L. was purchased from the local market, Giza, Egypt, in February 2012. The fruit was dehusked and washed, and uniform fruits without defects were selected by size and ripening stage (ripe stage = bright orange) [15, 16]. Then it was freeze dried and grounded to sawdust form, which was then kept in an air-tight brown bottle until use.

One hundred grams of fruitsawdust was soaked with 95% ethanol for two days with daily shaking and kept in refrigerator covered by a piece of aluminum foil. The extract was filtered with filter paper, while the residue was further extracted under the same conditions twice. The filtrates collected from three separate extractions were combined, and the filtrate was centrifuged at 3000 rpm for 10 min, then the ethanol was evaporated using a rotary evaporator apparatus attached with vacuum pump. The 100 gm of dried powder of* Physalis peruviana* L. yields 23% g.

### 2.2. Phytochemical Screening and Determination of Phenols and Flavonoids in* Physalis peruviana* L. Ethanolic Extract

The crude ethanolic extract of the fruit was subjected to qualitative chemical screening for the identification of the various major classes of active chemical constituents such as phenols, flavonoids, glycosides, phytosterols, saponins, tannins, and alkaloids using standard procedures of analysis [17, 18]. The quantity of extract total phenolic compounds was determined using a colorimetric method with Folin-Ciocalteu reagent [19] and was expressed as milligrams of gallic acid equivalent per gram of dry weight (mg GAE/g DW). The total flavonoid compounds in the extract was estimated using a colorimetric method [20] and was calculated as quercetin equivalent per gram dry weight (mg QE/g DW). All analyses were run in triplicate.

### 2.3. Laboratory Animals and Experimental Design

Thirty-six adult male Sprague-Dawley rats (weighing 180 ± 5 g) were purchased from the laboratory animal colony, Ministry of Health and Population, Helwan, Cairo, Egypt. All rats were provided with food and water* ad libitum* and all rats were fed on AIN 93 [21], in which soya bean oil was replaced with corn oil, throughout the experimental period. The experiment was carried out in accordance with the guidelines of the experimental animal ethics.

After 7 days of acclimatization, PPE was administered by oral gavage to the second and fifth groups at 100 mg/kg and third and sixth groups at 150 mg/kg once a day for 32 days. The dose of PPE has been chosen based on the previous [22].

To induce ARI, the fourth, fifth, and sixth groups were once intraperitoneally injected at day 28 with 5mg/kg of cisplatin, cis-diammineplatinum II dichloride, 1 mg/mL, Sigma, USA, according to previous studies [23, 24]. The normal control group and the second and the third groups were once intraperitoneally injected with saline at day 28. During the experimental period, all animals were weighed to monitor changes and to adjust the dosages of PPE accordingly. To ensure the incidence of ARI, blood samples were obtained from the orbital plexus of the normal control group and cisplatin control group (72 h after cisplatin injection). The mean values of creatinine and urea were significantly higher in cisplatin group than in normal control group (0.62 ± 0.03 versus 2.95 ± 0.43 mg/dL) and (23.21 ± 1.20 versus 109.32 ± 4.11 mg/dL).

### 2.4. Biochemical Analysis

At the end of the experimental period (on the fifth day after the cisplatin or saline injection), all animals were fasted overnight and were sacrificed under anesthesia with diethyl ether. Blood samples were collected from the aortic vein into clean dry centrifuge tubes and were centrifuged for 15 min at 3000 rpm to separate serum. Serum was carefully aspirated and transferred into dry clean Wasserman and kept frozen at −20°C till analysis. All kits used for biochemical analysis were obtained from the Egyptian American company for laboratory service and supplied by Alkan company, Dokki, Giza, Egypt.

Creatinine and urea were estimated according to Henry and Patton and Grouch, respectively [25, 26], while blood urea nitrogen was calculated by the following equation: BUN = serum urea (mg/dL)/2.14.

Total protein and albumin were determined according to Henry and Doumas et al., respectively [27, 28], while total globulin was calculated [29].

### 2.5. Kidney Tissue Examinations

The kidneys of each rat were removed immediately after sacrificing the animal, excised, rinsed, blotted dry with tissue paper, and weighed. Small portions of the right kidney were fixed in 10% neutral phosphate-buffered formalin and then processed for routine histological examination with hematoxylin-eosin staining.

The left kidney was homogenized in cold KCl solution (1.5%) to give a 10% homogenate. The homogenate was centrifuged at 10,000 g for 20 min at 4°C. The resultant supernatant was used for the determination of lipid peroxidation by thiobarbituric acid-reactive substances (TBARS) [30], reduced glutathione (GSH) [31], catalase (CAT) [32], and superoxide dismutase (SOD) [33].

### 2.6. Statistical Analysis

The results were expressed as mean ± SD. The differences among means were analyzed through one way analysis of variance (ANOVA) followed by Duncan's post hoc analysis, and the *P* values ≤ 0.05 were considered significant. SPSS software version 16 was used for the statistical analysis.

## 3. Results and Discussion

The phytochemical investigation of the crude ethanolic extract of* physalis peruviana* L. (Table 1) revealed the presence of phenols, flavonoids, glycosides, sterols, saponins, tannins, and alkaloids. In addition, phenolic and flavonoid compounds in the extract were 71.22 ± 2.75 mg/g DW and 167.19 ± 4.34 mg/g DW, respectively. It is worth to notice that most of the phytochemicals found in crude PPE have antioxidant property. The amounts of phenols and flavonoids determined in the present study were slightly different from those reported by another study [34] in which total flavonoids represent 226.19 ± 4.15 mg/g and total phenols represent 100.82 ± 6.25 mg/g. Comparisons of the results are difficult because of differences in the fruit origin, extraction, and analytical techniques used in both studies.

The difference between mean initial weights of all experimental groups was insignificant at the beginning of the experiment (Table 2). Rats in PPE100 and PPE150 gained weight in the same pattern of normal control groups, while cisplatin group lost weight markedly after cisplatin intraperitoneal injection which represent 168.6 ± 4.4 g compared to normal control group (205.7 ± 6.7 g). The final body weights of cisplatin groups that were pretreated with PPE at 100 or 150 mg/kg BW were higher (*P* < 0.05) than their corresponding control representing 178.3 ± 3.6 and 180.5 ± 4.1 g, respectively. Treatment with single dose of cisplatin caused marked reduction in feed intake (7.12 ± 1.9 g/day) compared to normal control group (12.63 ± 2.1 g/day). Furthermore, the feed intake of rats in PPE100 and PPE150 groups with or without cisplatin injection had insignificant mean feed intake when compared with normal control group. The relative kidney weights of rats in PPE100 and PPE150 groups were not significant compared to normal control group. On the other hand, cisplatin injection caused a significant increase in kidney relative weight. Pretreatment with PPE at 150 mg/dL caused significant improvement in kidney relative weight compared to cisplatin group.

The results that cisplatin group showed decrease in body weight and feed intake following cisplatin administration were in good agreement with [35] which stated that the loss in body weight of rats after injection of cisplatin may be due to gastrointestinal toxicity and by reduced ingestion of food. In addition, other authors reported that the reduction in body weight following cisplatin administration is caused by polyuria resulting from tubular injury, which in turn leads to dehydration, although gastrointestinal toxicity may also contribute [36, 37]. Unfortunately, measuring urine volume in the present study was difficult to be done. The inhibition of the body weight loss by PPE was considered as a direct or indirect evidence of their efficacy on the cisplatin induced ARI. The increase in kidney relative weight after cisplatin injection is confirmed by other studies that kidney weight generally increased as a result of kidney swelling which commonly occurs as a result of cisplatin induced ARI [38, 39]. The inhibition of the kidney relative weight by PPE was considered as a direct evidence of their efficacy on the cisplatin induced ARI.

Administration of* physalis peruviana* L. extract at the two levels did not alter serum creatinine, urea, and BUN values when compared to normal control rats (Table 3). Cisplatin injection produced a marked derangement in the kidney function and led to a significant increase in the level of serum creatinine, urea, and BUN when compared to normal control animals. Administration of PPE at 100 or 150 mg/kg BW before cisplatin injection produced significant improvement in these parameters compared to cisplatin group. The best improvement was observed in PPE150 + cisplatin group. As shown in Table 4, no significant differences were observed between normal control group and groups treated with PPE at 100 or 150 mg/kg BW concerning serum total protein, albumin, and total globulin. Animals administered with cisplatin excreted low levels of these parameters when compared to normal control group (*P* < 0.05). Interestingly, pretreatment with PPE at 150 mg/kg BW followed by 100 mg/kg BW produced significant improvement in serum total protein, albumin, and total globulin compared to their corresponding control. It is worth to notice that these levels were comparable to the levels of normal control group.

In cisplatin group, the levels of serum creatinine, urea, and BUN were significantly increased and serum protein metabolism parameters were significantly decreased. These results indicated that renal function was severely impaired in ARI rats. It is worth to know that there are several mechanisms that contribute to renal dysfunction following exposure to cisplatin that include direct tubular toxicity in the form of apoptosis and necrosis that is mediated through inflammation, reactive oxygen species, calcium overload, phospholipase activation, depletion of reduced glutathione, inhibition of mitochondrial respiratory chain function, induction of apoptosis, and ATP depletion [40–43]. The present findings are consistent with previous reports [24, 44, 45].

Cisplatin treatment resulted in a significant increase in the TBARS and significant decreases (*P* < 0.05) in the kidney GSH, CAT, and SOD in comparison to normal control group (Table 5). In the light of this, several studies demonstrated that cisplatin induced acute nephrotoxicity is mediated by depletion of renal reduced glutathione and by impaired activity of catalase and superoxide dismutase as well as an increase in renal lipid peroxidation markers [24, 46]. Pretreatment with PPE extract before cisplatin resulted in a significant decrease in TBARS concentration and significant increments in GSH, CAT, and SOD in kidney homogenate samples when compared to cisplatin control group. At the dose of 150 mg/kg,* physalis peruviana* L. extract caused the best results regarding TBARS and CAT. The present results demonstrated that PPE extract has the potential to attenuate nephrotoxicity of cisplatin. Pretreatment with PPE could alleviate ARI through enhancing antioxidant defense mechanisms which reduce oxidative stress that accompany ARI induced by cisplatin as mentioned thereinafter.

Reactive oxygen species and inflammation were shown to be the primary cause of nephrotoxicity in cisplatin induced ARI [47–49], so any active components in* physalis peruviana* L. that can play a role as antioxidants or as anti-inflammation may be responsible for such renoprotective effect. This approach was previously proved by a significant number of studies that antioxidants and anti-inflammatory agents can mitigate cisplatin induced nephrotoxicity [50–52].* Physalis peruviana* L. extracts have been proved to have antioxidant activities [22] and anti-inflammatory activities [13]. It is worth to know that the antioxidant activity of* physalis peruviana* L. is not a property of a single photochemical compound, but the synergistic effect of different antioxidants exists which in turn could alleviate oxidative stress and improve directly or indirectly the biological and biochemical parameters and kidney histology in PPE pretreated groups.

In this context, a study found that the antioxidant activity associated with* physalis peruviana* L. is due to the high levels of polyphenols [9]. The present study estimated good amounts of total phenolic and flavonoid compounds in* physalis peruviana* L. extract. In this respect, significant amounts of phenolic and flavonoid compounds in* physalis peruviana* L. extracts were reported [34], while others considered the fruit of* Physalis peruviana* L. as a source of phenolic compounds in food [6, 9]. Furthermore, Singh et al., concluded that renoprotective effect of polyphenols can be ascribed to their potent ROS scavenging and metal chelating properties [1]. In the light of this, it was found that quercetin is the main polyphenols in* physalis peruviana* L., followed by myricetin and kaempferol [53]. Quercetin, for example, has various biological activities such as antioxidant [54, 55] and anti-inflammatory functions [56]. Recently, it was reported that quercetin prevented the nephrotoxic effect of cisplatin [57].

The present study detected phytosterols and saponin also in* physalis peruviana* L. extract. Recent observations from animal and human studies have demonstrated anti-inflammatory effects of phytosterols in addition to their well known effect on lowering cholesterol levels [58]. Several biological effects have been ascribed to saponins, among them to be antioxidants [59]. Moreover,* physalis peruviana* L. extracts contain many withanolide glycosides [60]. Withanolides are natural steroidal lactones produced mainly by plants in the Solanaceae that often have many health benefits such as anti-inflammatory activity [61].

**Figure 1:**
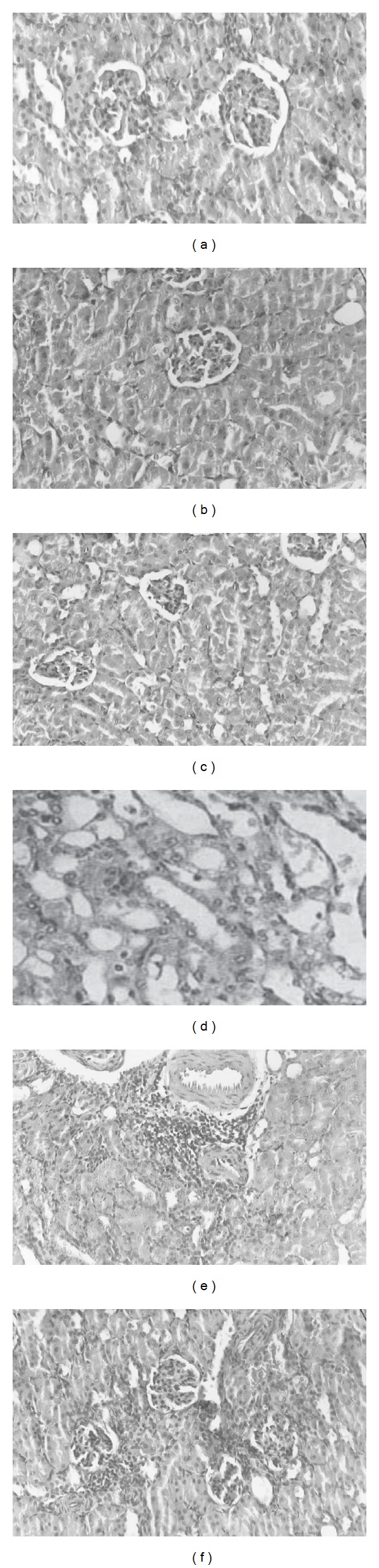
Effect of* Physalis peruviana* extract on the histology of kidney tissues in normal and cisplatin induced ARI rats. (a) Normal control; (b) 100 mg* physalis peruviana* extract/Kg BW; (c) 150 mg* physalis peruviana* extract/Kg BW; (d) cisplatin treatment alone; (e) pretreatment with 100 mg* physalis peruviana* extract/Kg BW+ cisplatin; (f), pretreatment with 150 mg* physalis peruviana* extract + cisplatin.

The kidney histology of both normal control group and groups treated with 100 or 150 mg PPE/Kg BW was found to be normal. In contrast, cisplatin caused marked injury with sloughing of tubular epithelial cells, loss of brush border, dilation of tubules, vacuolization, and intratubular cast formation (Figure 1). The previous changes were attenuated to some degree, very mild tubular necrosis and degeneration, in pretreated rats with 100 or 150 mg PPE/Kg BW. The improvement in kidney function as indicated by biochemical analysis was also reflected by less severe histological damage of the kidney. The morphological protection was better in ARI rats pretreated with 150 mg than 100 mg PPE/Kg BW.

In conclusion, Egyptian cape gooseberry (*physalis peruviana* L.) has a bright future as a functional food due to its high quality and quantity of nutrients and its bioactivities. The results of the present study indicated that pretreatment with PPE especially at 150 mg/kg BW significantly protected against the kidney injury induced by cisplatin. This protection may be mediated either by preventing the cisplatin-induced decline of renal antioxidant defense system or by the effect of its components. The active components and antioxidative mechanism(s) of action of PP ethanol extract and other forms of* physalis peruviana* L. warrant further studies in both* in vitro* and* in vivo* models.

## Figures and Tables

**Table 1:** Phytochemical screening of *Physalis peruviana * L. extract and its content of total phenols and flavonoids.

	*Physalis peruviana* L.
Phenols	+
Flavonoids	+
Phytosterols	+
Glycosides	+
Saponins	+
Tannins	+
Alkaloids	+
Extract phenols (mg/g DW)	71.22 ± 2.75
Extract flavonoids (mg/g DW)	167.19 ± 4.34

DW: dry weight; + indicates present. Values are expressed as mean ± SD.

**Table 2:** Effect of *Physalis peruviana  *L. extract on body weight, feed intake, and kidney relative weight in normal and cisplatin induced ARI rats.

	Initial Body weight (g)	Final body weight (g)	Feed intake (g/day/rat)	Kidney relative weight (%)
Normal control	180.0 ± 4.7^a^	205.7 ± 6.7^c^	12.63 ± 2.1^b^	0.73 ± 0.02^a^
PPE100	182.0 ± 2.6^a^	200.5 ± 4.3^c^	12.72 ± 1.2^b^	0.75 ± 0.05^a^
PPE150	180.0±4.1^a^	197.2 ± 3.7^c^	11.94 ± 2.5^b^	0.70 ± 0.09^a^
Cisplatin	181.0 ± 3.9^a^	168.6 ± 4.4^a^	7.12 ± 1.9^a^	1.32 ± 0.19^c^
PPE100 + cisplatin	183.0 ± 2.0^a^	178.3 ± 3.6^b^	10.51 ± 2.2^b^	0.93 ± 0.07^c^
PPE150 + cisplatin	183.0 ± 1.7^a^	180.5 ± 4.1^b^	10.93 ± 1.8^b^	0.85 ± 0.08^b^

Values are expressed as mean ± SD. Mean values within a column not sharing the same superscript letters were significantly different, *P* ≤ 0.05.

PPE100 and PPE150: 100 and 150 mg *Physalis peruviana  *L. extract/Kg BW, respectively.

**Table 3:** Effect of *Physalis peruviana  *L. extract on serum creatinine, urea, and BUN in normal and cisplatin induced ARI rats.

	Creatinine mg/dL	Urea mg/dL	BUN mg/dL
Normal control	0.65 ± 0.04^a^	23.33 ± 2.88^a^	10.90 ± 1.35^a^
PPE100	0.68 ± 0.02^a^	24.67 ± 2.31^a^	11.53 ± 1.08^a^
PPE150	0.66 ± 0.05^a^	22.27 ± 2.52^a^	10.59 ± 1.18^a^
Cisplatin	3.56 ± 0.81^d^	125.67 ± 5.03^d^	58.72 ± 1.77^d^
PPE100 + cisplatin	1.97 ± 0.04^c^	83.33 ± 3.51^c^	38.94 ± 1.64^c^
PPE150 + cisplatin	1.01 ± 0.34^b^	62.24 ± 2.08^b^	29.28 ± 0.97^b^

Values are expressed as mean ± SD. Mean values within a column not sharing the same superscript letters were significantly different. *P* ≤ 0.05; BUN: blood urea nitrogen. PPE100 and PPE150: 100 and 150 mg *Physalis peruviana  *L. extract/Kg BW, respectively.

**Table 4:** Effect of *Physalis peruviana  *L. extract on serum protein metabolism parameters in normal and cisplatin induced ARI rats.

	Total protein (g/dL)	Albumin (g/dL)	Total globulin (g/dL)
Normal control	7.39 ± 0.09^bc^	3.99 ± 0.47^b^	3.40 ± 0.23^b^
PPE100	7.18 ± 0.48^bc^	4.05 ± 0.57^b^	3.13 ± 0.17^b^
PPE150	6.99 ± 0.97^bc^	3.69 ± 0.32^b^	3.30 ± 0.30^b^
Cisplatin	5.01 ± 0.37^a^	2.25 ± 0.07^a^	2.76 ± 0.19^a^
PPE100 + cisplatin	6.78 ± 0.54^b^	3.29 ± 0.14^b^	3.49 ± 0.31^b^
PPE150 + cisplatin	7.22 ± 0.21^c^	3.82 ± 0.72^b^	3.20 ± 0.25^b^

Values are expressed as mean ± SD. Mean values within a column not sharing the same superscript letters were significantly different at *P* ≤ 0.05.

PPE100 and PPE150: 100 and 150 mg *Physalis peruviana  *L. extract/Kg BW, respectively.

**Table 5:** Effect of *Physalis peruviana  *L. extract on the kidney lipid peroxidation and antioxidant enzymes levels in normal and cisplatin induced ARI rats.

	TBARS nmol/g tissue	GSH *μ*mol/g tissue	CAT U/g tissue	SOD U/g tissue
Normal control	24.51 ± 1.99^a^	4.11 ± 0.75^c^	49.94 ± 2.75^c^	11.34 ± 0.49^c^
PPE100	22.76 ± 3.09^a^	4.82 ± 0.43^c^	50.63 ± 2.54^c^	12.35 ± 1.16^c^
PPE150	21.33 ± 2.11^a^	4.97 ± 0.59^c^	51.99 ± 2.65^c^	12.11 ± 0.52^c^
Cisplatin	48.86 ± 4.65^d^	2.75 ± 0.39^a^	24.13 ± 1.97^a^	5.23 ± 0.69^a^
PPE100 + cisplatin	37.54 ± 3.87^c^	3.91 ± 0.33^b^	37.34 ± 2.32^b^	8.96 ± 1.23^b^
PPE150 + cisplatin	30.11 ± 2.98^b^	3.99 ± 0.73^b^	42.09 ± 1.66^c^	9.99 ± 1.07^b^

Values are expressed as mean ± SD. Mean values within a column not sharing the same superscript letters were significantly different, *P* ≤ 0.05. PPE100 and PPE150: 100 and 150 mg *Physalis peruviana  *L. extract/Kg BW, respectively. TBARS: Thiobarbituric acid reactive substances; GSH: reduced glutathione; CAT: catalase; SOD: superoxide dismutase.
